# Robotic-Assisted Revision Total Knee Arthroplasty for Minimal Bone Loss: A Step-by-Step Surgical Technique

**DOI:** 10.3390/jcm15051972

**Published:** 2026-03-04

**Authors:** Jaad Mahlouly, Alexander Antoniadis, Thibaut Royon, Arnaud Fischbacher, Julien Wegrzyn

**Affiliations:** Department of Orthopedic Surgery and Trauma, Lausanne University Hospital and University of Lausanne, 1005 Lausanne, Switzerlandjulien.wegrzyn@chuv.ch (J.W.)

**Keywords:** robotic-assisted TKA, revision TKA, aseptic loosening, functional alignment, short cemented stem

## Abstract

**Background**: Revision total knee arthroplasty (rTKA) is a technically demanding procedure that, when performed using mechanically aligned strategies, frequently relies on stems, augments, metaphyseal cones and constrained implants to restore knee alignment and stability. In carefully selected cases with preserved metaphyseal bone stock and competent collateral ligaments, robotic assistance allows a bone-preserving strategy in which alignment, joint line height, and soft-tissue balance are restored using conventional posterior-stabilized components with short cemented stems rather than higher invasive and constrained constructs. **Methods**: This technical note describes a step-by-step surgical workflow using the Mako robotic system (Stryker) to revise failed primary TKA associated with minimal metaphyseal bone loss to rTKA with conventional posterior-stabilized components and short cemented stems within a functional alignment framework. **Results**: The workflow integrates CT-based three-dimensional planning, registration on in situ implants, real-time gap assessment, and precise robotic bone preparation to correct deformity and to restore stability while minimizing additional bone resection. In this setting, limited tibial metaphyseal defects are managed with impacted autologous cancellous graft, and stable fixation is achieved with short cemented stems. **Conclusions**: This robotic-assisted approach is intended as a bone-preserving option for selected rTKA cases associated with minimal bone loss and as a conceptual bridge between robotic-assisted primary and conventional revision TKAs performed with mechanical techniques and alignments.

## 1. Introduction

Revision total knee arthroplasty (rTKA) is among the most challenging procedures in reconstructive knee surgery. Successful outcomes depend on meticulous preoperative planning, the accurate restoration of limb alignment and joint line height, and the stable fixation on healthy bone stock [1,2]. Conventional revision strategies emphasize the systematic exposure, implant removal, and reconstruction of bone defects using stems, augments, metaphyseal cones, and constrained implants to restore the mechanical axis and knee stability [1,2,3,4,5,6]. Approaches such as those proposed by Abdel and Della Valle further highlight the importance of extensile exposure to manage complex revisions safely [7]. As Laskin underscored in “Ten Steps to an Easier Revision TKA”, minimizing bone loss and restoring balance are key determinants in long-term success [8]. In addition, as described by Morgan-Jones et al., the fixation of the implants is planned along three anatomical zones (i.e., epiphysis/joint surface, metaphysis, and diaphysis), with durable constructs achieving the secure fixation in at least two of these three zones [9]. Conventional revision techniques to achieve mechanical alignment may require additional bone resection and compromise the remaining metaphyseal bone stock, particularly when modular stems, augments, and metaphyseal cones are required. This may limit further reconstructive options and increase the technical complexity of subsequent revisions.

Recent robotic-assisted systems have introduced new possibilities for an improved accuracy, reproducibility, and personalization in knee arthroplasty. Both imageless second-generation platforms and CT-based robotic-arm systems allow three-dimensional planning, the precise correction of deformities, and intraoperative gap assessments [10]. These features can be leveraged not only in primary arthroplasties but also in selected revision TKAs where the bone stock is preserved, allowing functional alignment principles instead of conventional mechanical alignment to optimize knee balance and biomechanics while limiting additional bone resection.

The goal of this technical note is to describe a robotic-assisted, bone-preserving workflow for the revision of failed primary TKAs with minimal metaphyseal bone stock loss using conventional posterior-stabilized components with short cemented stems. This concept builds on the bone-preserving principles previously detailed for the robotic conversion of medial unicompartmental knee arthroplasty (UKA) to TKA and aims to provide a “bridge” between robotic-assisted primary and conventional revision TKAs performed with mechanical techniques and alignments [11].

## 2. Surgical Technique

This protocol has been developed to enable a revision TKA workflow using advanced robotic assistance. The strategy emphasizes functional alignment, precise three-dimensional preoperative planning, and dynamic intraoperative adjustment based on the real-time assessment of limb alignment, gap balance, and soft-tissue tension in line with recently described robotic techniques [11]. This technical note describes a step-by-step surgical workflow and does not report a clinical case series or clinical outcomes.

### 2.1. Preoperative Planning

For each case, a hip–knee–ankle CT scan is obtained according to a standard lower-limb protocol and uploaded into the Mako robotic software (Mako 3.1—Stryker, Mahwah, NJ, USA). A specific metal artifact reduction algorithm is not used at the time of image acquisition. Instead, artifact reduction is performed afterwards at the reconstruction console, where both a conventional bone-kernel series and a metal-artifact-reduced series are created. Residual artifacts related to the in situ TKA components are anticipated and considered when choosing the bone regions that will subsequently be used for registration and checkpoint validation.

The three-dimensional CT reconstruction is reviewed to evaluate the bone stock, the component position, the global alignment of the limb, and the surrounding soft-tissue envelope. Particular focus is placed on the metaphyseal bone quality, the size and location of bone defects, and the expected competence of the collateral ligaments based on combined clinical and imaging findings.

Within this framework, preoperative planning aimed to define functional alignment targets that preserved metaphyseal bone and restored the joint line height as close as possible to its anatomical position.

### 2.2. Patient Positioning and Approach

The patient is placed in the supine position on the operating table without the use of a tourniquet. The previous midline incision is incorporated whenever possible, and a standard medial parapatellar arthrotomy is carried out to expose the knee. The patella is everted laterally with care to protect the extensor mechanism. Synovectomy and debridement of fibrous tissue are performed as necessary to improve visualization while respecting the collateral ligaments. Synovial and periprosthetic tissue samples are harvested for microbiological culture and histopathological analysis according to our institutional protocol. The limb is then secured in a stable position to allow smooth tracking of the robotic arm and unrestricted range-of-motion testing throughout intraoperative assessment.

### 2.3. Pins Placement and Landmark Registration

**Figure 1:**
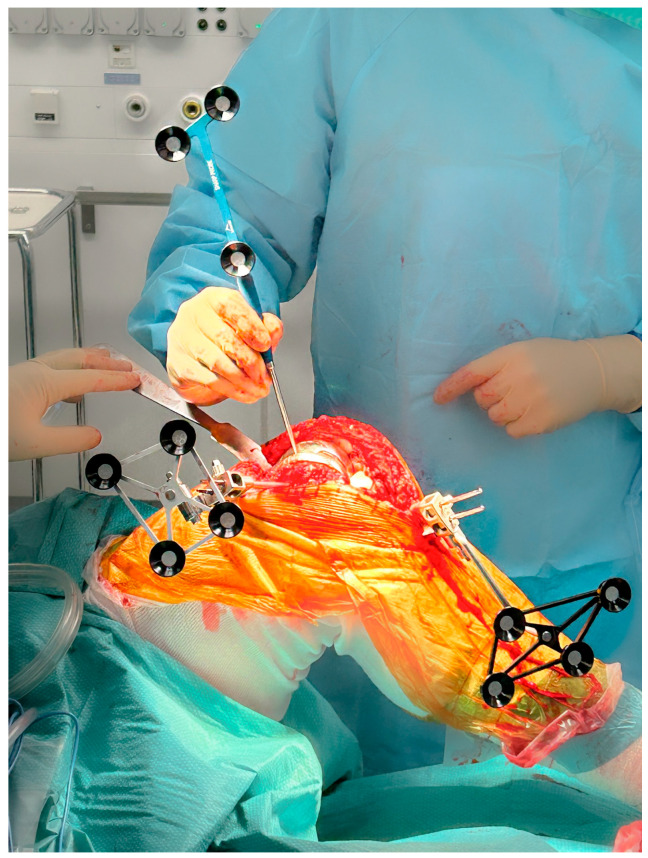
Intraoperative view after medial parapatellar approach reusing the previous incision, showing the primary total knee arthroplasty (TKA) in situ with femoral and tibial array pins in place while registration landmarks are being acquired directly from the metallic components.

Array pins for the robotic system (4 mm on the femoral side and 3 mm on the tibial side) are inserted according to the manufacturer’s protocol through the same medial parapatellar approach to minimize additional soft-tissue damage (Figure 1). The polyethylene insert is then removed to expose the relevant reference surfaces.

**Figure 2:**
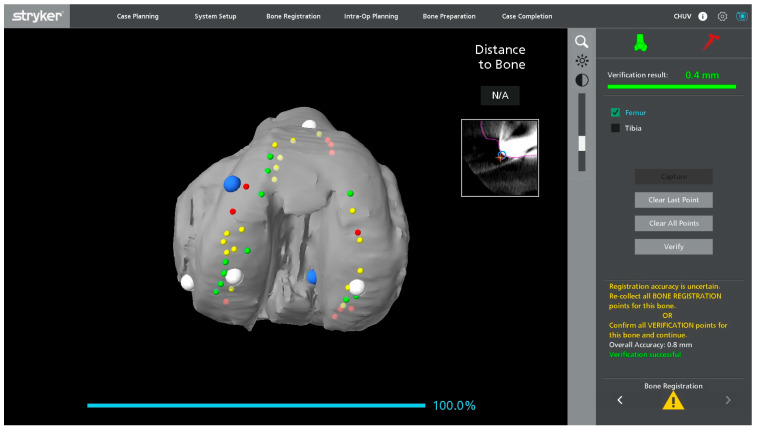
Screenshot of the robotic software showing registration landmarks on the virtual femoral component, with acquired and remaining target points displayed on the metallic surface.

**Figure 3:**
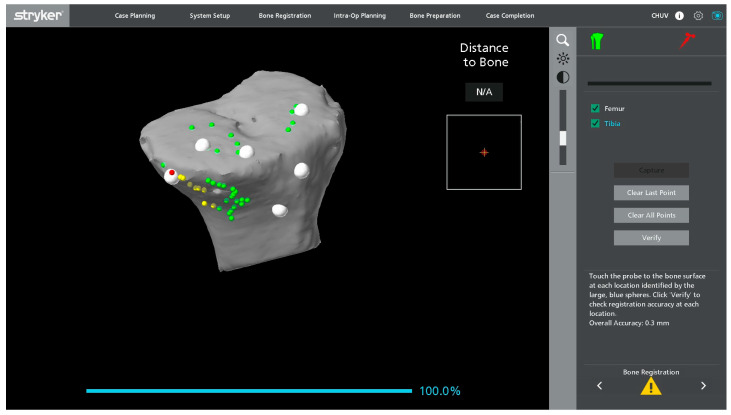
Screenshot of the robotic software showing registration landmarks on the virtual tibial component, with acquired and remaining target points displayed on the tray surface.

Registration landmarks are acquired directly from the in situ metallic femoral and tibial components as well as on the adjacent exposed bone (Figure 1). On the femoral side, registration is guided by the internal and external condylar contours, using a central condylar axis on the component to reduce the influence of metal artifacts on the CT and improve mapping accuracy (Figure 2). On the tibial side, points are collected over the central portion of the medial and lateral tibial plateau as well as on the metaphyseal bone (Figure 3).

Once landmark acquisition is complete, the polyethylene insert is reinserted to allow dynamic intraoperative assessment of ligament balance. In mobile-bearing designs, the definitive polyethylene insert can be reinserted. In fixed-bearing implants, particularly when the locking mechanism is damaged or uncertain, a trial insert can be used to avoid compromising the final component. Registration accuracy is verified using the system’s checkpoints. If any checkpoint shows a residual error greater than 0.5 mm, both landmarks and checkpoints are repeated until all values fall within the acceptable tolerance.

### 2.4. Intraoperative Alignment and Ligament Balance Assessment

**Figure 4:**
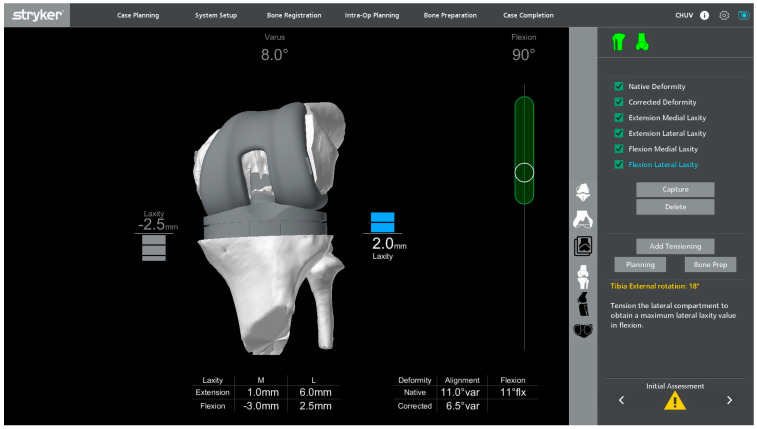
Initial intraoperative dynamic assessment with the primary total knee arthroplasty components and polyethylene insert in situ.

Once registration has been completed with the primary TKA components in situ, a first intraoperative assessment of functional alignment is carried out. In our technique, a functional alignment framework refers to using robot-assisted planning to optimize component positioning primarily through planned resections to achieve target gaps within predefined coronal and sagittal alignment boundaries. The targets include a hip–knee–ankle angle of approximately 180° ± 5° and a restoration of joint line height within ±2 mm of the native level. In revisions, the native joint line is estimated from available landmarks and radiographs with contralateral comparison when appropriate, and refined intraoperatively based on the gap balance. This planning also accounted for the restrictions imposed by short cemented stems due to potential cortical contact. Figure 4 illustrates a representative example of this initial assessment performed under varus–valgus stress, which may reveal coronal malalignment and gap asymmetry prior to plan adjustment. The polyethylene insert is reinserted so that the ligament behavior can be evaluated dynamically in flexion and extension. This can provide immediate feedback on the coronal and sagittal balance and help determine whether the initial plan requires adjustment. The limb is then taken through a full range of motion, and the robotic system is used to monitor the limb alignment and gap behavior in real time.

Varus–valgus stress is applied at different degrees of flexion to analyze the collateral ligament function and quantify the coronal laxity. The targets are the symmetric medial and lateral gaps in full extension. In flexion, a small lateral predominance is acceptable, with a lateral gap up to 2 mm larger than the medial gap at 90° of flexion, in keeping with the functional alignment principles and individualized gap planning [12].

**Figure 5:**
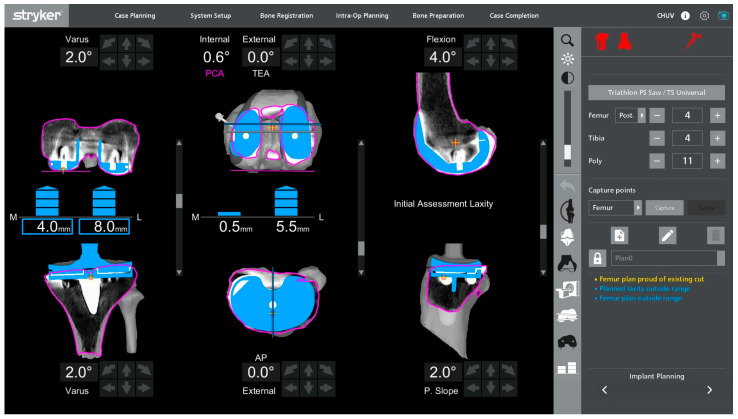
Screenshot of the Mako software showing the functional alignment plan with adjusted femoral and tibial resection levels on the three-dimensional CT-based reconstruction.

Under this functional alignment philosophy, the coronal and sagittal balance are primarily corrected by adjusting the component positioning and planned resection levels (Figure 5) rather than by performing extensive collateral ligament or soft-tissue releases. When adjusting the tibial coronal alignment, the varus–valgus position of the tibial baseplate must be planned while projecting the femoral and tibial 50 mm short stems, ensuring that their tips do not impinge on the metaphyseal cortical bone.

### 2.5. Implant Removal

After finalizing of the planification, the femoral and tibial components are removed using thin osteotomes, flexible saw blades, and dedicated extraction instruments. The careful sequential release of the bone–metal or bone–cement interface is performed while minimizing lever forces to reduce the risk of iatrogenic metaphyseal defects or fractures.

On the tibial side, particular care is given around the keel or stem region to preserve the remaining metaphyseal bone. All loose cement fragments and debris are removed under direct visualization. The femoral condyles, posterior cortex, and tibial plateaus are inspected to confirm that the bone stock has remained compatible with a bone-preserving revision strategy.

If a patellar button is present, well-fixed, and positioned without macroscopic wear lesions, no revision of this component is performed. In cases with a native patella, resurfacing is systematically performed during the rTKA to reduce the risk of subsequent anterior knee pain and potential reoperation.

### 2.6. Robotic Planning and Bone Resection

After implant removal, the exposed bone surfaces are compared with the preoperative plan. If the extent or pattern of bone loss differs from the preoperative assessment, the robotic plan is adjusted accordingly while maintaining the bone-preserving philosophy.

Robotic planning is restricted to the implants available in the robotic platform’s software library, and unsupported implant systems will require a conventional workflow.

Robotic-guided resections are then performed. Distal and posterior femoral resections are executed to restore the joint line height, posterior condylar offset, and flexion–extension gap symmetry. Tibial resection is limited to the minimum depth required to obtain a flat, stable platform, preserving as much metaphyseal bone as possible. The robotic system can provide real-time feedback on resection depth, orientation, and alignment, allowing for fine-tuning before completing each cut.

### 2.7. Bone Defect Management

After bone resections, residual contained metaphyseal defects are addressed. Autologous cancellous bone graft is harvested from the resected femoral or tibial bone, or supplemented with morselized allograft chips, and impacted into the defect with a tamp to recreate a stable and supportive bed for the cemented implants.

This technique is intended for defects that remain compatible with primary resections and a standard tibial baseplate footprint. Larger segmental defects, extensive uncontained bone loss, or situations requiring structural support beyond local grafting are considered outside the indications of this bone-preserving technique and warrant traditional revision constructs with stems, augments, and/or metaphyseal cones [13].

### 2.8. Final Implantation

**Figure 6:**
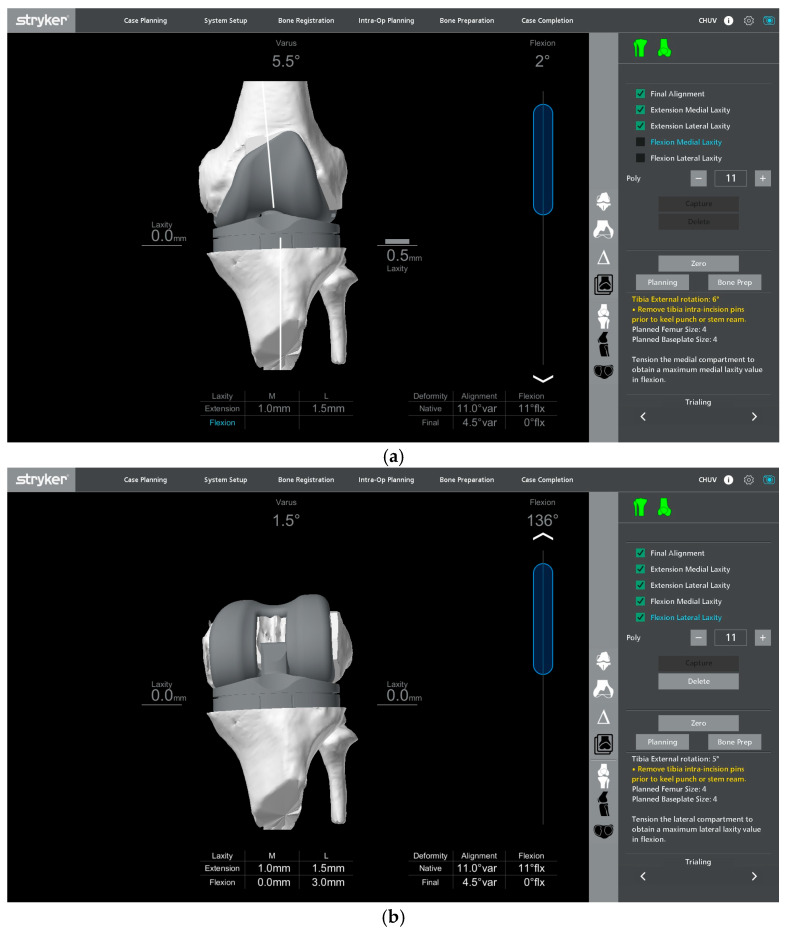
Screenshot of the robotic software showing intraoperative gap assessment with trial components in place under gentle varus–valgus stress, shown in full extension (**a**) and 90° flexion (**b**).

Trial components are inserted to confirm flexion–extension gap balance, stability, and patellar tracking. Alignment and soft-tissue tension are reassessed, both with and without varus–valgus stress in full extension and at 90° of flexion. The thickness of the polyethylene insert is adjusted as needed to achieve the planned balance according to the preplanned functional alignment strategy (Figure 6). The planned alignment, joint line position, and gap targets are achieved and confirmed intraoperatively using the robotic platform.

Once the surgeon is satisfied with the stability, alignment, and tracking, a definitive tibial preparation is performed, and definitive posterior-stabilized cemented components are implanted. Short 50 mm cemented stems are systematically used on both the tibial and femoral sides to enhance metaphyseal support while preserving the overall bone stock. The cement is pressurized on both the femoral and tibial sides to ensure adequate penetration into the prepared bone surfaces. The excess cement is removed, and the final component positions are checked both visually and with the robotic system’s verification tools.

The patella is then resurfaced as previously described.

### 2.9. Irrigation and Closure

The joint is irrigated thoroughly with saline and hemostasis is achieved. The arthrotomy is closed in a watertight fashion, followed by the closure of subcutaneous tissues and skin according to standard practice—no drain is routinely used. Postoperative care should follow an Enhanced Recovery After Surgery (ERAS) pathway [14]. A sterile dressing is applied for wound protection.

**Figure 7:**
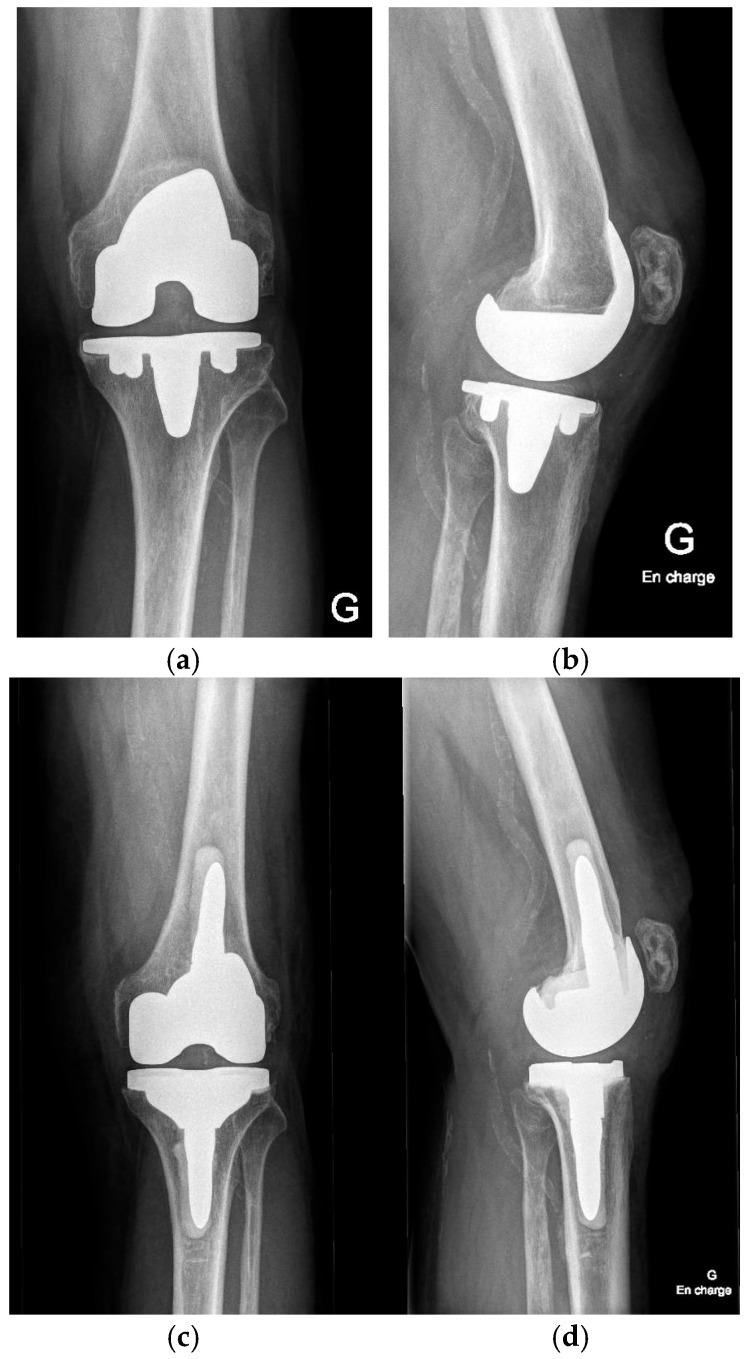
Radiographic comparison of preoperative (failed primary TKA due to aseptic loosening) and postoperative component positioning and alignment (**a**) preop AP view; (**b**) preop lateral view; (**c**) postop AP view; (**d**) postop lateral view.

Postoperatively, patients should follow a standardized rehabilitation program encouraging early mobilization and full weight-bearing as tolerated. Deep venous thrombosis prophylaxis and early quadriceps activation exercises are initiated according to institutional protocol. Postoperative radiographs of the knee and lower limb are obtained to confirm accurate component positioning (Figure 7).

## 3. Results

This described robotic workflow enabled stable registration on the in situ primary TKA components and facilitated controlled, bone-preserving implant removal. Functional alignment planning, combined with robotic guidance, corrected the preoperative deformity towards the planned alignment target within predefined coronal and sagittal boundaries and restored full range of motion. Gap behavior consistent with functional alignment principles was achieved without collateral ligament release or additional soft-tissue balancing procedures. The final femoral and tibial component positioning closely matched the preoperative 3D plan, and postoperative radiographs confirmed the restoration of limb alignment and appropriate component positioning.

## 4. Discussion

Revision TKA requires the accurate management of bone loss, implant fixation, soft-tissue balance, and stability. Classical revision concepts, as described by Dennis et al. and further refined by Abdel and Laskin, emphasized the systematic exposure, implant removal, restoration of the joint line, and reconstruction of bone defects using stems, augments, and constrained implants to restore alignment and stability [1,2,7,8]. While these strategies have yielded reliable outcomes, they often require substantial additional bone resection and may compromise the remaining metaphyseal bone stock, particularly in younger or more active patients [1,2,3]. In selected cases with limited bone defects that do not require metaphyseal cones or long stems, the zonal fixation framework described by Morgan-Jones et al. can support a bone-preserving strategy, provided that stable fixation can still be achieved in at least two of the three zones (epiphysis, metaphysis, diaphysis) [9].

Robotic assistance offers a potential paradigm shift in this setting. Cochrane et al. recently reported that robotic-assisted rTKA with a second-generation imageless system can achieve precise bone preparation and reproducible alignment with favorable early outcomes [10]. By integrating three-dimensional planning, controlled bone resections, and real-time gap assessment, robotic workflows can enhance intraoperative accuracy and standardization while maintaining the versatility to adapt to intraoperative findings. In parallel, bone-preserving CT-based robotic workflows have been described for UKA-to-TKA conversions, further supporting the role of robotic assistance in complex knee reconstruction [11].

In the present surgical technique, robotic assistance provided a controlled environment for applying the principles emphasized by Dennis and Laskin, namely to preserve the bone, restore the joint line, and ensure stability, within a functional alignment framework [1,2,8]. Fine-tuning of soft-tissue balance is achieved through dynamic intraoperative assessment rather than extensive collateral ligament releases, thereby supporting a bone-preserving strategy in selected cases with competent ligamentous structures.

The management of limited tibial metaphyseal defects follows the principles described by Huten et al., using impacted autologous cancellous graft beneath the tibial baseplate to restore a stable platform without resorting to stems or augments [3,12]. Current evidence summarized by Yapp et al., Ayekoloye et al., and Driesman et al. has indicated that cemented constructs and stemmed revision strategies can provide reliable mid- to long-term survival in complex rTKAs, and that cemented fixation remains a widely accepted option in this context [4,5,6]. In this case, the bone stock is preserved and the soft-tissue envelope is intact. Therefore, cemented fixation using standard posterior stabilized components is considered sufficient. For limited bone defects, the routine use of short 50 mm cemented stems can provide additional metaphyseal support. This approach is consistent with the Morgan-Jones zonal fixation concept because it can secure epiphyseal and metaphyseal fixation in two of the three zones without the need for metaphyseal cones or long diaphyseal stems [9].

Conceptually, this workflow extends the bone preserving robotic approach previously reported for UKA to TKA conversion to the setting of failed primary TKA [11]. The goal is to implement a functional alignment strategy with conservative bone resections using a standardized workflow. Alignment targets may be constrained by the use of 50 mm short cemented stems to avoid cortical tip contact. In appropriately selected patients, this approach also limits the routine need for augments or highly constrained implants.

This bone-preserving robotic workflow is intended for patients with a preserved metaphyseal bone stock (AORI Types I and IIA—with defects amenable to correction by primary resections and local grafting), competent collateral ligaments, and no clinical or radiological suspicion of periprosthetic joint infections. Cases with extensive bone loss, collateral ligament insufficiency or persistent instability throughout the arc of motion, suspected or confirmed infection, or severely compromised bone quality remain better suited to conventional revision-type constructs using stems, cones, augments, and higher levels of constraints [1,2,3,7]. If registration accuracy checkpoints are not met, the registration is repeated after addressing correctable causes. If an acceptable accuracy cannot be achieved, the robotic steps are discontinued and the procedure is completed using a conventional workflow.

This technical note is limited by its descriptive nature and by the absence of systematic clinical and radiographic outcome data, which will be reported in a future study. Furthermore, this workflow should be restricted to high-volume surgeons familiar with both robotic-assisted TKA and rTKA principles, as a learning curve should be anticipated for surgeons new to robotic platforms. The technique is more straightforward in designs with modular or easily removable mobile polyethylene inserts. In fixed-bearing constructs, care must be taken not to damage the insert during removal so that it can either be reinserted for intraoperative ligament balancing or use the trial component of the construct in place. In addition, when planning the tibial coronal alignment, the position and length of the tibial and femoral stems must be considered to avoid impingement on the metaphyseal cortical bone, particularly in the presence of asymmetric defects. Larger prospective series and comparative studies are needed to evaluate the reproducibility, efficiency, and survivorship of robotic-assisted bone-preserving revision workflows compared with conventional revision techniques [10,11].

## 5. Conclusions

This technical note outlines a bone-preserving robotic-assisted workflow to revise failed primary TKA associated with minimal metaphyseal bone loss to rTKA with conventional posterior-stabilized components and short cemented stems within a functional alignment framework. The procedure integrates three-dimensional CT-based planning, registration on in situ implants, functional alignment principles, and dynamic intraoperative ligament assessments with the aim of supporting standardized execution and intraoperative decision-making while preserving the metaphyseal bone.

This approach is intended for carefully selected patients with an adequate metaphyseal bone stock, competent collateral ligaments, and no evidence of periprosthetic joint infections, whereas remaining cases with extensive bone loss, ligament insufficiency, infection, or compromised bone quality can be more appropriately managed with conventional revision-type constructs. We recommend that this workflow be implemented in centers with established experience in robotic TKA and revision knee arthroplasty. A consecutive case series is underway to refine indications and evaluate the safety, effectiveness, and implant survivorship of this bone-preserving robotic-assisted revision strategy.

## Data Availability

No datasets were generated or analyzed. De-identified operative images underlying the figures are available from the corresponding author upon reasonable request, subject to institutional and patient-privacy restrictions.

## Abbreviations

The following abbreviations are used in this manuscript:

UKA	Unicompartmental Knee Arthroplasty
TKA	Total Knee Arthroplasty
rTKA	Revision Total Knee Arthroplasty
